# Factors associated with physical violence by a sexual partner among girls and women in rural Kenya

**DOI:** 10.7189/jogh.07.020406

**Published:** 2017-12

**Authors:** Deborah A Gust, Yi Pan, Fred Otieno, Tameka Hayes, Tereza Omoro, Penelope A Phillips–Howard, Fred Odongo, George O Otieno

**Affiliations:** 1 Centers for Disease Control and Prevention, Division of HIV/AIDS Prevention, Atlanta, Georgia, USA; 2 Nyanza Reproductive Health Society, Kisumu, Kenya; 3 ICF, Atlanta, Georgia, USA; 4 Kenya Medical Research Institute, Kisumu, Kenya; 5 Department of Clinical Sciences, Liverpool School of Tropical Medicine, Liverpool, UK

## Abstract

**Background:**

Intimate partner physical violence increases women’s risk for negative health outcomes and is an important public health concern. The purpose of the present study was to determine 1) the proportion of girls (≤18 years) and women (>18 years) who experienced physical violence by a sexual partner, and 2) factors (including self–reported HIV infection) associated with girls and women who experienced physical violence by a sexual partner.

**Methods:**

Cross–sectional surveys conducted in the Gem Health and Demographic Surveillance System (HDSS) area in Siaya County, western Kenya in 2011–2012 (Round 1) and 2013–2014 (Round 2).

**Findings:**

Among 8003 unique participants (582 girls and 7421 women), 11.6% reported physical violence by a sexual partner in the last 12 months (girls: 8.4%, women: 11.8%). Three factors were associated with physical violence by a sexual partner among girls: being married or cohabiting (nearly 5–fold higher risk), low education, and reporting forced sex in the last 12 months (both with an approximate 2–fold higher risk). Predictive factors were similar for women, with the addition of partner alcohol/drug use and deliberately terminating a pregnancy. Self–reported HIV status was not associated with recent physical violence by a sexual partner among girls or women.

**Conclusions:**

Gender–based physical violence is prevalent in this rural setting and has a strong relationship with marital status, low education level, and forced sex among girls and women. Concerted efforts to prevent child marriage and retain girls in school as well as implementation of school and community–based anti–violence programs may help mitigate this risk.

Intimate partner violence (IPV), where an individual (mostly, but not exclusively, a male) causes physical, sexual, or emotional abuse to their partner, occurs worldwide and has serious physical health and psychological consequences [1]. Moreover, IPV against women can contribute to an increased risk for HIV directly through forced sex or indirectly, for example, through power inequity in negotiating condom use [2], or an increase in risk behaviors such as substance abuse [3]. In addition, women who experience IPV may not receive needed health care, including regular HIV testing [4]. An analysis of the 2008–2009 Demographic and Health Survey [5] in Kenya found a significant association between IPV and HIV infection, controlling for sociodemographic and other risk factors [6]. Importantly, studies have found that culturally–based gender inequalities, including power and resource distribution, contribute to an environment that fosters high levels of IPV and HIV infection [7].

Adolescent girls are at a greater risk of HIV infection compared to their male age–mates in lower and middle income countries [8]. Our aim was to better understand the dynamics of risk for physical violence (eg, hitting, slapping, kicking) by a sexual partner and associated factors among girls separately from women. Our setting was Siaya County, western Kenya, where HIV prevalence is high (estimated 24.8% among persons 15–49 years of age) [9]. Specifically, the purpose of the present study was to determine 1) the proportion of girls (≤18 years of age) and women (>18 years of age) who experienced physical violence by a sexual partner, and 2) factors (including self–reported HIV status) associated with girls and women who experienced physical violence by a sexual partner.

## METHODS

### Design

Two cross–sectional surveys evaluating HIV risk behaviors, HIV sero–status factors, HIV prevention services and care and treatment uptake were conducted within the Kenya Medical Research Institute (KEMRI) Health and Demographic Surveillance System (HDSS) area in Gem, located in Siaya County in rural western Kenya. The KEMRI HDSS offered a sampling frame of all the registered housing compounds (14 501 in 2010). A compound is a cluster of houses usually occupied by households of the same extended family and usually demarcated by a fence. A random sample of 4000 compounds was selected through a community–based simple random approach. This entailed all compounds in the village being given a unique registration number, thus there was no possibility of sampling bias.

Compounds were randomly chosen by a combination of methods: community participation which included community leaders (n = 25) picking pieces of paper from a bucket and the study statistician picking random numbers via a computer until a total sampling frame of approximately 6000 compounds was identified. Trained field staff visited each compound; all households within the compound were approached and all individuals aged 13 years and above who had slept there the prior night and gave informed consent were interviewed. All eligible persons were verbally informed of the study, and if in agreement then provided written consent (see Ethics section). The two surveys took place March 2011 to September 2012, and January 2013 to April 2014, respectively. The survey targeted 15 000 individuals. Persons who were not residents of the HDSS, but who slept in the sampled compound the night prior to the survey date, were also enrolled and interviewed as long as they met the inclusion criteria and provided informed consent. The intention was to interview the same persons in the sampled housing compounds during Round 1 and Round 2 in order to develop a community–based platform to evaluate various infectious and non–infectious disease interventions. However due to in–migrations and out migrations experienced in the HDSS, not all respondents in Round 1 were interviewed in Round 2 and vice versa, as well as people simply not found at home.

### Study location and population.

HDSS was launched in September 2001 by the US Centers for Disease Control and Prevention in collaboration with KEMRI, and provides general demographic and health information, as well as disease and intervention information, in western Kenya. Gem HDSS is located about 20 km northeast of Lake Victoria in Siaya County, formerly Siaya District. Residents are predominately of Luo ethnicity, and their major economic activity is subsistence farming [10]. The mid–year population in Gem in 2012 was 86 279 across 21 131 households grouped by extended families into 14 954 compounds. Females comprised approximately half (52.5%) of the population [11]. The area experiences substantial in and out migration, in part due to young men seeking employment in the nearby city of Kisumu and beyond, and the traditional exogamous marriage system where persons marry outside of the group to which they belong.

### HIV risk behavioral survey

Using the Gem HDSS as a sampling frame, a questionnaire focusing on HIV risk behaviors and health service uptake was administered immediately before HIV testing and counseling to avoid knowledge of the HIV test result which could have influenced responses. The questionnaire targeted all persons in the household (males and females) meeting specific age criteria. Inclusion criteria were age ≥13 years, resident of Gem, and willing to give informed consent to participate in the survey. The questionnaire was structured and pre–coded and administered using a computer–assisted personal interview (CAPI). This was similar to a paper questionnaire because trained interviewers asked participants the questions and recorded their responses. Thus, even the few who were functionally illiterate could participate in the survey. The questionnaire was available in three languages, English, Kiswahili and Dholuo. Trained field staff used the HDSS household list to randomly select eligible household members for an interview. All interviews were conducted in the home privately. Interviewers were trained on the general interviewing techniques as well as on how to use Questionnaire Design Studio (QDS) for CAPI.

### Measures

The dependent variable was female responses to the question, “Have any of your sexual partners in the last year hit/slapped/kicked or done anything else to hurt you physically in the last 12 months?” (herein referred to as “experienced physical violence by a sexual partner”) (yes/no). It should be noted that emotional abuse was not captured in this question. Independent variables were chosen from the survey based on the existing literature and included age, highest level of education, source of income, marital status (henceforth married also includes cohabiting), number of pregnancies, ever deliberately terminated a pregnancy, lifetime number of sex partners, ever used a condom (yes/no), age at first sex, most recent self–reported HIV test result, would say yes/no to sex if they knew their partner had an STI, forced sex in last 12 months (yes/no) (refers to same last 12 months as physical violence by a sexual partner), and partner used alcohol before last sex and/or drug use in last 12 months (yes/no). For the last 3 independent variables, if the response was “yes” for any of the sexual partners the respondent had in the last 12 months, the variable was coded as a “yes”. Because a large number of participants had missing HIV laboratory test results, we used self–reported HIV status. To ensure that self–reported HIV status was a valid measure, we examined a subset of persons in Round 1 who had both laboratory verified HIV test result and self–reported test results for degree of agreement. The kappa statistic was 0.87, which indicated that self–reported HIV status could be used as a substitute for the laboratory reported HIV result.

### Ethics

All persons received information about the objectives of the study and were informed that the information they provided would be kept private, that they could choose not to participate, and that they would not be identified when the information was reported. Girls 13–17 years of age provided consent if they were independent mature minors (living with a consensual sexual partner, currently pregnant or already a mother). For non–mature minors, parental consent and child assent was required. Thus, informed consent or assent was obtained from all individual participants included in the study. Survey participants were given a bar of soap as a token of appreciation for their participation. All procedures performed were in accordance with the ethical standards of the institutional and national research committee and with the 1964 Helsinki declaration and its later amendments or comparable ethical standards. The study protocol, consent forms, and data collection instruments were reviewed and approved by the Centers for Disease Control and Prevention and the KEMRI local and national Ethical Review Committees (SSC1801).

### Analysis

We conducted descriptive analyses as well as bivariate and multiple regression analyses to determine factors associated with girls (≤18 years old) and women (>18 years old) who experienced physical violence by a sexual partner. Due to sparse numbers, we had to combine the responses to two questions regarding partner use of alcohol and partner use of drugs even though the time frames were different. We collapsed the data for number of pregnancies because there were not enough females with zero pregnancies to be able to code nulliparous as a separate category. In the first step of model selection, a bivariate model was applied which included only a single variable at a time to be associated with experiencing physical violence by a sexual partner. All candidate variables that had *P*–values ≤0.2 in the bivariate model were entered into the multivariable model selection. In developing a final multivariable model, backward selection was applied with a *P*–value less than 0.05 as the selection criterion. A robust Poisson model with sandwich standard error estimator (generated by generalized estimating equation [GEE]) was used to estimate the risk ratios (RR) in the bivariate models and adjusted risk ratios (aRR) in the multivariable models [12,13].

The intent was to re–interview the same persons in Round 1 and Round 2 and to interview persons in the sampled compounds in Round 2 who were not found during Round 1. However, due to immigration and emigration, some persons who participated in the Round 1 survey were not available to participate in the Round 2 survey and persons were interviewed in Round 2 who did not participate in Round 1. Thus, we used GEE to control for the correlation between the two rounds of data. For the females who participated in Round 1 and Round 2, if the female reported having experienced physical violence by a sexual partner in Round 1, in Round 2 or in both, they were considered as having experienced physical violence by a sexual partner.

## RESULTS

**Figure 1:**
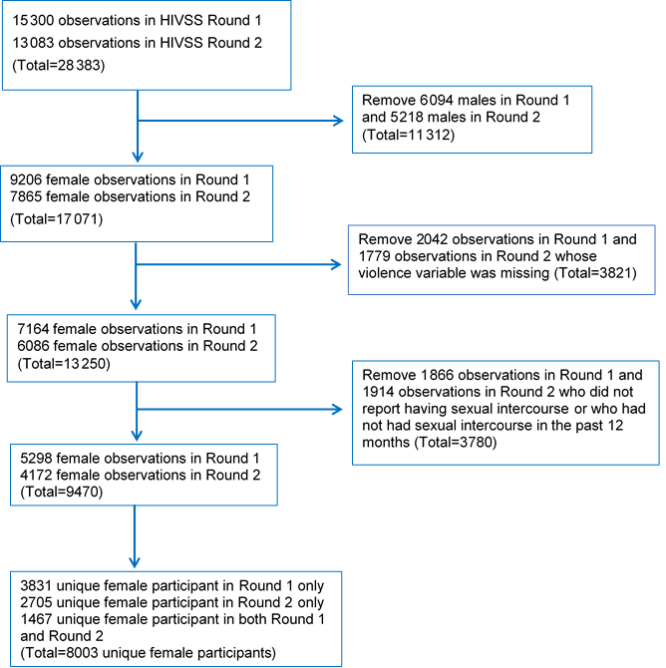
Derivation of female participants ≤18 years and >18 years of age from the two rounds of the HIV substudy from the Health and Demographic Surveillance System, a population registration system that monitors health and demographic dynamics, Gem, Kenya, 2011–2012 (Round 1) and 2013-2014 (Round 2).

There were a total of 28 383 participants surveyed during the two study rounds, of whom 11 312 were male and thus removed from the analysis. Among the females, 3821 were removed because they did not answer the violence question. Finally, as we were interested in examining experience of physical violence by a sexual partner, we removed 3780 females who did not report ever having had sexual intercourse or who had not had sexual intercourse in the past 12 months. After accounting for duplicates (the same person interviewed in Rounds 1 and 2), the final number of unique participants was 8003 (**Figure 1**). Most women participated in only one of the two Rounds of data collected (Round 1 = 3831; Round 2 = 2705). There were 1467 females who participated in both Round 1 and Round 2.

The median age of the survey participants in our analysis was 31 (interquartile range (IQR) = 23, 42). The median age was 17 years old for girls in Round 1 with IQR (16, 18) and the same for Round 2. The median age was 32 years for women in Round 1 with IQR (25, 43) and also 32 years in Round 2 with IQR (25, 44). Nearly half of girls ≤18 years of age and more than 85% of women >18 years of age were married (**Table 1**). The youngest participant who reported being married was 13 years of age. Among 13–year–old participants in Round 1, 16.7% (2 of 12) were married and in Round 2, 14.3% (1 of 7) were married. Data in **Table 1** are based on total unique participants. The proportion of females who self–reported being HIV–positive was 13.5% in Round 1 (girls: 3.2%, women: 14.3%) and 15.2% in Round 2 (girls: 3.6%, women 15.8%).

**Table 1:** Characteristics of female participants in Gem, Kenya, 2011–2012 (Round 1) and 2013–2014 (Round 2)*

	Round 1 (N = 5298)			Round 2 (N = 4172)		
	**Total**	**≤18 y old (n = 393)**	**>18 y old (n = 4905)**	**Total**	**≤18 y old (n = 223)**	**>18 y old (n = 3949)**
**Median age in years (IQR)**	31 (23, 42)	17 (16, 18)	32 (25, 43)	31 (24, 43)	17 (16, 18)	32 (25, 44)
**Highest level of education (n, %):**						
None	394 (7.4)	1 (0.3)	393 (8.0)	275 (6.6)	1 (0.5)	274 (6.9)
Primary incomplete and complete	3999 (75.5)	307 (78.1)	3692 (75.3)	3167 (75.9)	180 (80.7)	2987 (75.6)
Secondary incomplete and complete	826 (15.6)	83 (21.2)	743 (15.2)	663 (15.9)	42 (18.8)	621 (15.7)
Tertiary	79 (1.5)	2 (0.5)	77 (1.6)	67 (1.6)	0 (0.0)	67 (1.7)
**Sources of income (cash) (n, %):**						
None	669 (12.6)	98 (25.0)	571 (11.6)	783 (18.8)	77 (35.3)	706 (17.9)
Work/Business	3728 (70.4)	105 (26.7)	3623 (73.9)	2714 (65.2)	48 (22.0)	2666 (67.6)
Partner/Family	770 (14.5)	185 (47.1)	585 (11.9)	655 (15.7)	92 (42.2)	563 (14.3)
Other	130 (2.5)	5 (1.3)	125 (2.6)	11 (0.3)	1 (0.5)	10 (0.3)
**Marital status (n, %):**						
Single	478 (9.0)	198 (50.4)	280 (5.7)	395 (9.5)	123 (55.2)	272 (6.9)
Married	4391 (82.9)	192 (48.9)	4199 (85.6)	3474 (83.3)	98 (44.0)	3376 (85.5)
Divorced/Widowed/Separated	429 (8.1)	3 (0.8)	426 (8.7)	303 (7.3)	2 (0.9)	301 (7.6)
**Number of pregnancies (n, %):**						
0	324 (6.1)	143 (37.1)	181 (3.7)	322 (7.7)	89 (40.3)	233 (5.9)
1–2	1320 (25.0)	225 (58.3)	1095 (22.4)	1013 (24.4)	125 (56.6)	888 (22.5)
3–4	1291 (24.4)	6 (1.6)	1285 (26.3)	1044 (25.1)	6 (2.7)	1038 (26.4)
5 or more	2347 (44.4)	12 (3.1)	2335 (47.7)	1781 (42.8)	1 (0.5)	1780 (45.2)
**Deliberately terminated a pregnancy (n, %):**						
Yes	317 (6.4)	15 (6.3)	302 (6.4)	171 (4.4)	8 (6.0)	163 (4.4)
No	4623 (93.6)	223 (93.7)	4400 (93.6)	3678 (95.6)	125 (94.0)	3553 (95.6)
**Lifetime number of sexual partners (n, %):**						
1	762 (15.5)	150 (38.9)	612 (13.5)	746 (19.8)	86 (39.1)	660 (18.6)
2	1426 (29.0)	122 (31.6)	1304 (28.8)	1280 (34.0)	76 (34.6)	1204 (34.0)
3 or more	2730 (55.5)	114 (29.5)	2616 (57.7)	1739 (46.2)	58 (26.4)	1681 (47.4)
**Ever used a condom (n, %):**						
Yes	1794 (33.9)	247 (62.9)	1547 (31.6)	1414 (33.9)	141 (63.2)	1273 (32.3)
No	3498 (66.1)	146 (37.2)	3352 (68.4)	2753 (66.1)	82 (36.8)	2671 (67.7)
**If you knew your partner had an STI would say no to sex (n, %):**						
Yes	4507 (85.8)	323 (82.8)	4184 (86.1)	3152 (76.3)	162 (75.4)	2990 (76.4)
No	745 (14.2)	67 (17.2)	678 (13.9)	978 (23.7)	53 (24.7)	925 (23.6)
**Forced sex last 12 months (n, %):**						
Yes	1129 (21.3)	63 (16.0)	1066 (21.8)	368 (8.8)	20 (9.0)	348 (8.8)
No	4165 (78.7)	330 (84.0)	3835 (78.3)	3801 (91.2)	203 (91.0)	3598 (91.2)
**Partner alcohol or drug use at last sex (last 12 months) (n, %):**						
Yes	887 (18.0)	36 (10.0)	851 (18.6)	497 (12.0)	8 (3.6)	489 (12.5)
No	4045 (82.0)	325 (90.0)	3720 (81.4)	3648 (88.0)	213 (96.4)	3435 (87.5)
**Age at first sex (n, %):**						
≤15 years	2660 (50.2)	121 (30.8)	2539 (52.0)	2320 (55.6)	78 (35.0)	2242 (56.8)
>15 years	2683 (49.8)	272 (69.2)	2366 (48.2)	1852 (44.4)	145 (65.0)	1707 (43.2)
**Self–reported HIV status (n, %):**						
Positive	645 (13.5)	11 (3.2)	634 (14.3)	605 (15.2)	7 (3.6)	598 (15.8)
Negative	4127 (86.5)	338 (96.9)	3789 (85.7)	3381 (84.8)	187 (96.4)	3194 (84.2)

*Out of 9470 girls and women surveyed, there were 8003 unique participants; 1467 took part in both Round 1 and Round 2 data collection.

Among the 8003 unique participants, a significantly higher proportion of women reported physical violence by a sexual partner than girls (girls: 8.4%, women: 11.8%; *P* = 0.01). Of the 3831 females who participated only in Round 1, 8.6% of girls and 14.7% of women experienced physical violence from a sexual partner. Of the 2705 females who participated only in Round 2, 7.4% of girls and 10.0% of women experienced physical violence from a sexual partner. Lastly, of the 1467 females who participated in both data collection rounds, 25.0% (2 of 8) of girls and 8.2% (120 of 1459) of women experienced physical violence by a sexual partner.

### Bivariate analysis: Factors associated with girls who experienced physical violence by a sexual partner in the last year

The risk of experiencing physical violence by a sexual partner was twice as high for girls who had no education or only some primary schooling (RR = 2.04, 95% confidence interval (CI) = 1.17, 3.56) compared to girls who had completed primary school or had higher levels of education. The risk was nearly 5 times as high for girls who were married or cohabiting (RR = 4.73, 95% CI = 2.43, 9.19) compared to being single, divorced widowed or separated, and nearly 5 times as high for girls who had 3 or more lifetime sexual partners (RR = 4.74, 95% CI = 2.23, 10.09) compared to 1. In addition, the risk of experiencing physical violence by a sexual partner was approximately twice as high for girls who reported their partners never used a condom (RR = 2.05, 95% CI = 1.23, 3.43), and would not say no to sex if partner had an STI (RR = 2.18, 95% CI = 1.29, 3.70). Finally, the risk was more than twice as high among girls who reported forced sex in last 12 months (RR = 2.52, 95% CI = 1.45, 4.38) and that their partner used alcohol at last sex and/or drugs in last 12 months (RR = 2.60, 95% CI = 1.36, 4.99). The number of girls who experienced physical violence by a sexual partner and self–reported being HIV positive (n = 3) were too few for analysis (**Table 2**).

**Table 2:** Factors associated with females who experienced physical violence by a sexual partner in the last 12 months by age; Gem, Kenya, 2011–2012 (Round 1) and 2013–2014 (Round 2)*

	≤18 years				>18 years			
**Variables**	**Experienced physical violence by a sexual partner**				**Experienced physical violence by a sexual partner**			
	**Yes**	**No**	**RR (95% CI)**	***P***	**Yes**	**No**	**RR (95% CI)**	***P***
**Highest level of education:**			–	0.01			–	0.02
None or some primary	36 (67.9)	281 (49.9)	2.04 (1.17, 3.56)		621 (57.9)	4200 (54.0)	1.15 (1.03, 1.29)	
Primary or above	17 (32.1)	282 (50.1)	Ref		451 (42.1)	3582 (46.0)	Ref	
**Sources of income (cash):**				0.2				<0.0001
None	11 (20.8)	164 (29.4)	Ref		107 (10.0)	1170 (15.0)	Ref	
Job/business	19 (35.9)	134 (24.0)	1.94 (0.95, 3.97)	0.07	786 (73.4)	5503 (70.8)	1.51 (1.25, 1.83)	<0.0001
Partner/family/other	23 (43.4)	260 (46.6)	1.32 (0.66, 2.64)	0.4	178 (16.6)	1105 (14.2)	1.65 (1.32, 2.07)	<0.0001
**Marital status:**				<0.0001				<0.0001
Single/Divorced/Widowed/Separated/ Other	10 (18.9)	316 (56.1)	Ref		85 (7.9)	1194 (15.3)	Ref	
Married/Cohabiting	43 (81.1)	247 (43.9)	4.73 (2.43, 9.19)		987 (92.1)	6588 (84.7)	1.94 (1.57, 2.40)	
**Number of pregnancies:**				–				<0.0001
0–2	50 (96.2)	532 (95.9)	–		311 (29.0)	2086 (26.9)	1.29 (1.12, 1.49)	0.0004
3–4	2 (3.9)	10 (1.8)	–		351 (32.8)	1972 (25.4)	1.51 (1.31, 1.72)	<0.0001
≥5	0 (0.0)	13 (2.3)	Ref		409 (38.2)	3706 (47.7)	Ref	
**Deliberately terminated a pregnancy:**				0.5				<0.0001
Yes	5 (11.1)	18 (5.5)	0.65 (0.22, 1.99)		111 (10.8)	354 (4.8)	2.07 (1.74, 2.46)	
No	40 (88.9)	308 (94.5)	Ref		918 (89.2)	7035 (95.2)	Ref	
**Lifetime No. of sexual partners:**				0.0001				<0.0001
1	8 (15.7)	228 (41.1)	Ref		117 (11.4)	1155 (16.4)	Ref	
2	15 (29.4)	183 (33.0)	2.21 (0.96, 5.08)	0.06	273 (26.7)	2235 (31.7)	1.18 (0.96, 1.46)	0.1
≥3	28 (54.9)	144 (26.0)	4.74 (2.23, 10.09)	<0.0001	633 (61.9)	3664 (51.9)	1.60 (1.33, 1.95)	<0.0001
**Ever used a condom:**				0.006				0.02
Yes	24 (45.3)	364 (64.7)	Ref		376 (35.1)	2444 (31.5)	Ref	
No	29 (54.7)	199 (35.4)	2.05 (1.23, 3.43)		696 (64.9)	5327 (68.6)	0.87 (0.77, 0.98)	
**If you knew your partner had an STI would say no to sex:**				0.004				0.1
Yes	34 (65.4)	451 (81.6)	Ref		850 (79.8)	6324 (82.0)	Ref	
No	18 (34.6)	102 (18.4)	2.18 (1.29, 3.70)		215 (20.2)	1388 (18.0)	1.12 (0.98, 1.29)	
**Forced sex in last 12 months:**				0.001				<0.0001
Yes	15 (28.3)	68 (12.1)	2.52 (1.45, 4.38)		427 (39.9)	987 (12.7)	3.43 (3.07, 3.82)	
No	38 (71.7)	495 (87.9)	Ref		644 (60.1)	6789 (87.3)	Ref	
**Partner used alcohol before last sex and/or drug use in last 12 months:**				0.004				<0.0001
Yes	9 (17.3)	35 (6.6)	2.60 (1.36, 4.99)		3331 (32.3)	1009 (13.5)	2.49 (2.21, 2.81)	
No	42 (82.4)	496 (93.4)	Ref		694 (67.7)	6461 (86.5)	Ref	
**Age at first sex:**				0.70				<0.0001
≤15 years	16 (30.2)	183 (32.5)	Ref		512 (47.8)	4269 (52.9)	Ref	
>15 years	37 (69.8)	380 (67.5)	1.11 (0.63, 1.95)		560 (52.2)	3513 (49.1)	1.27 (1.14, 1.42)	
**Self–reported HIV status:**				–				
Positive	3 (6.4)	15 (3.0)	–		163 (16.5)	1069 (14.8)	1.12 (0.95, 1.31)	0.2
Negative	44 (93.6)	481 (97.0)	Ref		826 (83.5)	6157 (85.2)	Ref	
**Round:**				0.7				<0.0001
1	35 (66.0)	358 (63.6)	1.12 (0.65, 1.93)		704 (65.7)	4201 (54.0)	1.54 (1.38, 1.73)	
2	18 (34.0)	205 (36.4)	Ref		368 (34.3)	3581 (46.0)	Ref	

RR – risk ratio; 95% CI – confidence interval; Ref – reference value

*Percentage in each cell is computed using the total number of non–missing records as the denominator. In some cases the respondents had missing data for the covariates.

### Multivariable analysis: Factors associated with girls who had experienced physical violence by a sexual partner in the last year

In the final multivariable analysis, the risk of physical violence by a sexual partner was nearly twice as high among girls who had no education or only some primary school (aRR = 1.98, 95% CI = 1.15, 3.41) compared to girls who had completed primary school or had higher levels of education, nearly 5 times as high among girls who were married or cohabiting (aRR = 4.67, 95% CI = 2.42, 8.98) compared to being single, divorced widowed or separated, and more than twice as high among girls who reported forced sex (aRR = 2.39, 95% CI = 1.39, 4.14) (**Table 3**).

**Table 3:** Multivariable analysis – factors associated with experiencing physical violence by a sexual partner in the last 12 months by girls ≤18 years old, Gem, Kenya, 2011–2012 (Round 1) and 2013–2014 (Round 2)

Variables	aRR (95% CI)	*P*
**Highest level of education:**		0.01
None or some primary	1.98 (1.15, 3.41)	
Primary or above (Ref)	–	
**Marital status:**		<0.0001
Single/Divorced/Widowed/Separated (Ref)	–	
Married/Cohabiting	4.67 (2.42, 8.98)	
**Forced sex last 12 months:**		
Yes	2.39 (1.39, 4.14)	0.002
No (Ref)		

aRR – adjusted risk ratio; 95% CI – confidence interval, Ref – reference value

### Bivariate analysis: Factors associated with women who experienced physical violence by a sexual partner in the last year

The risk of experiencing physical violence by a sexual partner was 15% higher for women who had no education or only some primary school (RR = 1.15, 95% CI = 1.03, 1.29) compared to women who had completed primary school or had higher levels of education. The risk was nearly 50% higher for women who had income from a job or business (RR = 1.51, 95% CI = 1.25, 1.83) or partner/family (RR = 1.65, 95% CI = 1.32, 2.07) than no source of income, nearly twice as high for women who were married or cohabiting (RR = 1.94, 95% CI = 1.57, 2.40) compared to being single, divorced widowed or separated, and 29% higher for women who had 0–2 (RR = 1.29, 95% CI = 1.12, 1.49) and 51% higher for women who had 3–4 pregnancies (RR = 1.51, 95% CI = 1.31, 1.72) compared to those having 5 or more. In addition, the risk was twice as high for women who deliberately terminated a pregnancy (RR = 2.07, 95% CI = 1.74, 2.46), 60% higher for women who had 3 or more lifetime sexual partners (RR = 1.60, 95% CI = 1.33, 1.95) compared to 1, and over three times as high for women who reported forced sex in last 12 months (RR = 3.43, 95% CI = 3.07, 3.82). Finally, the risk of experiencing physical violence by a sexual partner was more than twice as high among women who reported that their partner used alcohol at last sex and/or drugs in the last 12 months (RR = 2.49, 95% CI = 2.21, 2.81), 27% higher among women who reported age at first sex was >15 years (RR = 1.27, 95% CI = 1.14, 1.42) and 13% lower among women who reported their partners had not ever used a condom (RR = 0.87, 95% CI = 0.77, 0.98). The risk of experiencing sexual violence by a partner was 54% higher among women in Round 1 (RR = 1.54, 95% CI = 1.38, 1.73) than in Round 2. There was no significant association between women who experienced physical violence by a sexual partner and self–report of HIV status (RR = 1.12, 95% CI = 0.95, 1.31) (**Table 2**).

### Multivariable analysis: Factors associated with women who experienced physical violence by a sexual partner in the last year

In the final multivariable analysis, the risk of experiencing physical violence by a sexual partner was 16% higher among women who had no education or only some primary school (aRR = 1.16, 95% CI = 1.03, 1.30) compared to women who had completed primary school or had higher levels of education and twice as high for women who were married or cohabiting (aRR = 2.04, 95% CI = 1.60, 2.59) compared to being single, divorced widowed or separated. In addition, the risk was 34% higher among women who had ≥3 lifetime sexual partners (aRR = 1.34, 95% CI = 1.10, 1.63) compared to 1 and more than 2 times as high among women who reported forced sex in last 12 months (aRR = 2.53, 95% CI = 2.23, 2.88). The risk was 62% higher among women who had 0–2 pregnancies (aRR = 1.62, 95% CI = 1.39, 1.88) and 55% higher among women who had 3–4 pregnancies (aRR = 1.55, 95% CI = 1.36, 1.78) compared to those having 5 or more. Finally, the risk was nearly twice as high among women who reported that their partner used alcohol at last sex and/or drugs in the last 12 months (aRR = 1.82, 95% CI = 1.61, 2.08) and 45% higher among women who reported deliberately terminating a pregnancy (aRR = 1.45, 95% CI = 1.21, 1.74). The risk of experiencing physical violence by a sexual partner was 21% higher among women in Round 1 (aRR = 1.21, 95% CI = 1.07, 1.37) than in Round 2 (**Table 4**).

**Table 4:** Multivariable analysis – factors associated with being experiencing physical violence by a sexual partner in the last 12 months by women >18 years old in Gem, Kenya, 2011–2012 (Round 1) and 2013–2014 (Round 2)

Variables	aRR (95% CI)	*P*
**Highest level of education:**		0.01
None or some primary		
Primary or above (Ref)	1.16 (1.03, 1.30)	
**Marital status:**		<0.0001
Single/Divorced/Widowed/ Separated/Other (Ref)		
Married/Cohabiting	2.04 (1.60, 2.59)	
**Lifetime No. of sexual partners:**		0.002
1 (Ref)		
2	1.08 (0.87, 1.34)	0.50
≥3	1.34 (1.10, 1.63)	0.004
**Forced sex last 12 months:**		<0.0001
Yes	2.53 (2.23, 2.88)	
No (Ref)		
**Number of pregnancies:**		<0.0001
0–2	1.62 (1.39, 1.88)	<0.0001
3–4	1.55 (1.36, 1.78)	<0.0001
≥5 (Ref)		
**Partner alcohol use at last sex and/or drug use in last 12 months:**		<0.0001
Yes	1.82 (1.61, 2.08)	
No (Ref)		
**Deliberately terminated a pregnancy:**		
Yes	1.45 (1.21, 1.74)	<0.0001
No (Ref)		
**Round:**		
1	1.21 (1.07, 1.37)	0.002
2 (Ref)		

aRR – adjusted risk ratio; 95% CI – confidence interval

## DISCUSSION

We found that nearly 12% of females in Gem, rural western Kenya, reported experiencing physical violence by a sexual partner in the last 12 months with the proportion significantly lower for girls (8.4%) than for women (11.8%). This is on the lower end of the range of reports of any physical violence (not just from a sexual partner) in the last year of between 3% and 52% of women in a global review [14] and lower than the 48.5% of Kenyan girls aged 13 to 17 years statistics [5]. Though, this is similar to the proportion (10%) reported in a study of pregnant Kenyan women seeking antenatal care [15]. While some study results have shown a relationship between IPV and HIV infection [6,16], like some other studies [15], we did not find an association. This may be due to different populations and cultures studied as well as the differences in study methods and variables measured [6].

Girls and women had three factors associated with experiencing physical violence by a sexual partner in common: low education, being married or cohabiting, and experiencing forced sex in the last 12 months. The risk of experiencing physical violence by a sexual partner was nearly twice as high for girls who had no education or only some primary school education compared to girls who had completed primary school or higher. For women the risk was lower, though nonetheless significant with women who had less education having a 15% greater risk of experiencing physical violence by a sexual partner than women with primary school education and above. Low education level has repeatedly been shown to be a risk factor for a host of adverse life events experienced by girls and women ranging from early sexual initiation [17], sexually transmitted diseases, abuse [18], HIV infection [19], mortality [20] and early marriage and pregnancy [17]. Relatedly, early marriage and pregnancy are important factors with regard to school drop–out [21]. Our study showed that physical violence by a sexual partner was nearly five times as high for girls and twice as high for women who were married or cohabiting. The fact that being married or cohabiting was strongly associated with physical violence by a sexual partner is not unexpected given the girl or woman’s extended period of exposure to the sexual partner compared to those single, divorced or widowed. Finally, experiencing physical violence by a sexual partner was more than twice as high for girls and women who reported forced sex. It is noteworthy that forced sex is reported to be most often perpetrated by intimate partners [22]. In rural western Kenya, 41% of married girls aged 14 to 19 years reported forced sex by their spouses, while 45% reported physical abuse by their spouses [23]. While physical violence and forced sex are both types of intimate partner or domestic violence, we did not combined them as we were interested in correlates of physical violence. This separation of different types of intimate partner or domestic violence, is used by the United States Department of Justice [24]. Lack of education is interconnected with early marriage and forced sex. The 2004 Global Campaign for Education [25] report states: “it is general schooling that appears to make the most powerful impact on young people’s sexual behavior and choices. A complete primary education leads to increased ability to evaluate, understand and apply facts; gains in confidence; and greater decision–making power in relationships” (p. 7). Other research has shown how multiple issues, such as food insecurity, illiteracy and poverty [26], interact with gender inequalities like forced sex [27].

Other factors associated with women who experienced physical violence by a sexual partner included alcohol and/or drug use by their partners and ever deliberately terminating a pregnancy. Data from the Kenya Demographic Health Survey identified Nyanza Province to have the highest rate of IPV with alcohol the strongest risk factor, specifically, a 2.5–higher rate of IPV if husbands are often drunk compared with non–drinking husbands [5]. In a meta–analysis of intimate partner violence and pregnancy termination, while experiencing IPV was not always a factor in wanting to end a pregnancy, the analysis suggested that violence can lead to a pregnancy (via coercion, rape, sexual assault, or contraceptive sabotage) which is then terminated [28].

Our study had several limitations. First, we only included questions on physical violence; we did not include questions on emotional or verbal abuse. Other studies have suggested an overlap between physical violence and psychological and sexual violence [24,29]. Second, we did not include a question on lifetime experience with physical violence from a sexual partner, only violence in the last 12 months. Third, we used questions that were not validated and relatedly, used only a single measure, not a violence scale. Fourth, HIV status was based on self–reported results and the lack of a relationship between physical violence by a sexual partner and HIV may have been affected by the fact that only those aware of their status were included in the analysis. Fifth our analysis was cross-sectional so the direction of associations cannot be determined. Sixth, the variables partner alcohol use at last sex and drug use in the last 12 months had small numbers so had to be combined. Seventh, some potentially important variables were not included in our analysis due to omission (eg, age at marriage or age at which cohabitation was initiated). Eighth, there was a potential for some causality between Round 1 and Round 2; interventions that occurred between the two survey rounds may have played a role. Finally, there may have been social desirability bias; our results may be low due to under reporting of physical abuse by a sexual partner.

In conclusion, low education level, being married or cohabiting, and reporting forced sex in the last 12 months were common factors associated with physical violence by a sexual partner for both girls and women in rural Kenya. Addressing violence against women is an urgent public health need [30]. To change the circumstances and conditions that can lead to violent behavior, we advocate focusing on keeping children in school at least through secondary school to reduce early marriage, to allow the development of characteristics such as dependability, judgement, motivation, and effort [31]. Areas of future research may include anti–violence interventions aimed at both girls and boys as a way to change cultural norms as well as wellness interventions aimed at families to support parental efforts to provide both formal education and wellness skills to their children that can also help in preventing early marriage. There is a convergence of global attention on the importance of social and structural factors impacting health with programs such as “Let Girls Learn”, which builds on the USAID campaign for girls’ education [32], “Together for Girls” which stimulates policies and programs to prevent sexual violence and provides supportive care and services for victims of sexual violence [33] and the DREAMS (Determined, Resilient, Empowered, AIDS–Free, Mentored and Safe) initiative, which aims to identify what is putting adolescent girls and young women at risk for HIV in 10 high burden countries in Africa [34] as well as multiple interventions [35,36]. For women and girls not in school, community structural and educational programs are needed, for instance a microfinance and training intervention, has been shown to reduce levels of intimate–partner violence [37], in addition to safe places to seek support. The World Health Organization suggests increasing access to post–primary, vocational and technical education for women to prevent IPV and improve overall health [38]. Indeed, Freudenberg and Ruglis [39] state “Education is one of the strongest predictors of health: the more schooling people have, the better their health is likely to be” (p. 1). This one intervention of keeping girls as well as boys in school through at least secondary school, with teachers trained in non–violent discipline [40] and tailored programs on anti–violence and personal, economic and sexual empowerment, should be a major focus of public health.
